# Supplementary material for: The Expansion of the Spectrum in Stuttering Disorders to a Novel ARMC Gene Family (ARMC3)
Source: Genes (Basel). 2022 Dec 6;13(12):2299. doi: 10.3390/genes13122299 (PMC9778410; doi:10.3390/genes13122299)
Supplement: Supplementary file 1 [file genes-13-02299-s001.zip › genes-2028962-supplementary.pdf]

Supplementary Materials

Table S1. *In silico* interpretations of ARMC3, BCHE and CACNA1F variants.

Gene	Variant	S. No.	Pathogenicity Prediction Tools	Scores	Prediction status	ACMG 2015	Co-segregation
ARMC3	c.916+1G>A	1	MutationTaster	1	Disease causing	Pathogenic of Uncertain Significance (PVS1, PP3, BS1)	Yes
		2	PolyPhen-2	NA	NA		
		3	SIFT	NA	NA		
		4	PROVEAN	NA	NA		
		5	VarSome	5/6	Pathogenic		
		6	CADD	28.8	Deleterious		
		7	VarSEAK	13.81%	Splicing Effect		
		8	RegSNP-Intron	0.82	Damaging		
		9	Human Splice Finder	-137.71%	Most probably affecting splicing		
		10	MaxEntScan	--------------	Likely disruptive		
		11	FATHMM-MKL	0.9567	Pathogenic		
		12	EIGEN	0.8194	Pathogenic		
		13	EIGEN PC	0.5837	Pathogenic		
		14	BayesDel noAF	-0.0014	Pathogenic		
		15	PhyloP100way	5.724	Highly conserved		
		16	GERP	5.7699	Conserved,		
BCHE	c.293A>G; p.Asp98Gly	1	MutationTaster	0.9999	Disease causing	Pathogenic (PP5, PS3, PM5, BP4)	No
		2	PolyPhen-2	0.852	Possibly damaging		
		3	SIFT	0.05	Pathogenic		
		4	PROVEAN	5.56	Pathogenic		
		5	VarSome	14/17	Pathogenic		
		6	CADD	23.7	Deleterious		
		7	VarSEAK	NA	NA		
		8	RegSNP-Intron	NA	NA		
		9	Human Splice Finder	NA	NA		
		10	MaxEntScan	NA	NA		
		11	FATHMM-MKL	0.98567	Pathogenic		
		12	EIGEN	0.3421	Uncertain		
		13	EIGEN PC	0.3432	Uncertain		
		14	BayesDel noAF	0.2349	Pathogenic		
		15	PhyloP100way	7.226	Highly conserved		
		16	GERP	--------------	Conserved		
CACNA1F	c.1555G>A; p.Gly519Ser	1	MutationTaster	0.99951	Disease causing	NPF	No
		2	PolyPhen-2	0.999	Probably damaging		
		3	SIFT	0.01	Damaging		
		4	PROVEAN	NPF	NPF		
		5	VarSome	NPF	NPF		
		6	CADD	24.3	Deleterious		
		7	VarSEAK	NA	NA		
		8	RegSNP-Intron	NA	NA		
		9	Human Splice Finder	NA	NA		
		10	MaxEntScan	NA	NA		
		11	FATHMM-MKL	NPF	NPF		
		12	EIGEN	NPF	NPF		
		13	EIGEN PC	NPF	NPF		
		14	BayesDel noAF	NPF	NPF		
		15	PhyloP100way	7.819	Highly conserved		
		16	GERP	--------------	Conserved		

Abbreviations: NA; Not applicable, NPF; No prediction found

Table S2. The identified variants causing persistent developing stuttering in the previous and present studies.

Gene	OMIM	Chr.	Cytogenic location	cDNA change	Amino acid change	Exon	dbSNP	Variant type	Mutation type	Familial/Sporadic	Origin	References
GNPTAB	607840	12	q23.2	c.961A>G	p.Ser321Gly	9	rs137853824	SNV	Missense	Familial	Pakistani	[17]
				c.1363G>T	p.Ala455Ser	11	rs137853822					
				c.1875C>G	p.Phe624Leu	13	rs137853823					
				c.3598G>A	p.Glu1200Lys	19	rs137853825					
GNPTG	607838	16	p13.3	c.11_19dup	p.Leu5_Arg7dup	1	rs1195696340	Duplication	Duplication	Familial	Pakistani	[17]
				c.74C>A	p.Ala25Glu	2	rs137853826	SNV	Missense			
				c.688C>G	p.Leu230Val	9	rs137853827					
NAGPA	607985	16	p13.3	c.252C>G	p.His84Gln	2	rs755458782	SNV	Missense	Familial	Pakistani	[17]
				c.982C>T	p.Arg328Cys	6	rs139526942					
				c.1538_1553del	p.Phe513Serfs*113	10	NA	Deletion	Frameshift			
AP4E1	607244	15	q21.2	c.1549G>A	p.Val517Ile	14	rs760021635	SNV	Missense	Familial	Cameroonian	[18]
				c.2401G>A	p.Glu801Lys	18	rs556450190					
IFNAR1	107450	21	*q22.11*	c.1282A>C	p. Lys428Gln	9	rs563741878	SNV	Missense	Familial	Chinese	[13]
				c. 1655T>C	Leu552Pro	11	rs762410025			Familial	Chinese	
				c. 902G>A	p. Gly301Glu	7	rs375386475			Sporadic	Chinese	
				c.1002_1004delTCC	p. Pro335del	8	rs72552343	Deletion	Deletion	Sporadic	Chinese	
ARMC3	611226	10	p12.2	c.916+1G>A	NA	Splice donor site of intron-8	rs767509621	SNV	Splice site	Familial	Pakistani	**Present study**

Abbreviations: Chr; Chromosome, SNV; Single nucleotide variant, NA; Not applicable
